# Delirium, Caffeine, and Perioperative Cortical Dynamics

**DOI:** 10.3389/fnhum.2021.744054

**Published:** 2021-12-20

**Authors:** Hyoungkyu Kim, Amy McKinney, Joseph Brooks, George A. Mashour, UnCheol Lee, Phillip E. Vlisides

**Affiliations:** ^1^Department of Anesthesiology, University of Michigan Medical School, Ann Arbor, MI, United States; ^2^Center for Consciousness Science, University of Michigan Medical School, Ann Arbor, MI, United States; ^3^Neuroscience Graduate Program, University of Michigan, Ann Arbor, MI, United States

**Keywords:** postoperative cognitive changes, electroencephalography, delirium, cognitive aging, cognitive dysfunction

## Abstract

Delirium is a major public health issue associated with considerable morbidity and mortality, particularly after surgery. While the neurobiology of delirium remains incompletely understood, emerging evidence suggests that cognition requires close proximity to a system state called *criticality*, which reflects a point of dynamic instability that allows for flexible access to a wide range of brain states. Deviations from criticality are associated with neurocognitive disorders, though the relationship between criticality and delirium has not been formally tested. This study tested the primary hypothesis that delirium in the postanesthesia care unit would be associated with deviations from criticality, based on surrogate electroencephalographic measures. As a secondary objective, the impact of caffeine was also tested on delirium incidence and criticality. To address these aims, we conducted a secondary analysis of a randomized clinical trial that tested the effects of intraoperative caffeine on postoperative recovery in adults undergoing major surgery. In this substudy, whole-scalp (16-channel) electroencephalographic data were analyzed from a subset of trial participants (*n* = 55) to determine whether surrogate measures of neural criticality – (1) autocorrelation function of global alpha oscillations and (2) topography of phase relationships *via* phase lag entropy – were associated with delirium. These measures were analyzed in participants experiencing delirium in the postanesthesia care unit (compared to those without delirium) and in participants randomized to caffeine compared to placebo. Results demonstrated that autocorrelation function in the alpha band was significantly reduced in delirious participants, which is important given that alpha rhythms are postulated to play a vital role in consciousness. Moreover, participants randomized to caffeine demonstrated increased alpha autocorrelation function concurrent with reduced delirium incidence. Lastly, the anterior-posterior topography of phase relationships appeared most preserved in non-delirious participants and in those receiving caffeine. These data suggest that early postoperative delirium may reflect deviations from neural criticality, and caffeine may reduce delirium risk by shifting cortical dynamics toward criticality.

## Introduction

Delirium reflects an acute, fluctuating disturbance in cognition and awareness that affects 20-50% of older surgical patients (Dasgupta and Dumbrell, 2006; Avidan et al., 2017). Delirium during surgical recovery is associated with increased mortality (Lisk et al., 2020), cognitive and functional decline (Saczynski et al., 2012; Hshieh et al., 2017), and prolonged hospitalization (Gleason et al., 2015). Furthermore, delirium shares pathophysiologic overlap with Alzheimer’s Disease-Related Dementias (Fong et al., 2019), and patients experiencing delirium demonstrate increased risk of subsequent dementia (Witlox et al., 2010; Sprung et al., 2017). There is thus a need to improve neurobiological understanding of delirium to inform diagnostic and therapeutic strategies. Furthermore, given the pathophysiological overlap between delirium and major neurocognitive disorders, such an advanced understanding may also help to identify patients at risk for future cognitive impairment.

Emerging evidence suggests that cognition requires dynamic coordination of neural activity across space and time (Alderson et al., 2020). Because delirium is characterized by impairment of executive functions, delirium may also reflect breakdown of coordinated neural activity that supports cognitive function. To achieve such integration of activity, one theory holds that the brain positions itself near critical phase transitions, such that brain states can flexibly shift, as needed, for meeting demands (Hesse and Gross, 2014). This property illustrates the concept of *criticality*, which refers to the state of a system when it is poised at the boundary of a phase transition - from an ordered to a more disordered state (Shew and Plenz, 2013). With *neural* criticality, the brain is postulated to reside at such a so-called critical state, which enables dynamic state transitions that may facilitate cognition (Hesse and Gross, 2014). For example, neural network computation is optimized when residing near the point of criticality (Bertschinger and Natschläger, 2004), suggesting that critical dynamics support information processing. Indeed, functional magnetic resonance imaging (MRI) data suggest that such dynamic state transitions underlie cognitive performance in healthy volunteers (Alderson et al., 2020). Given that delirium reflects impaired cognitive functions (e.g., attention), disruptions in neural criticality may plausibly contribute to delirium. However, the association between critical dynamics and delirium has not been previously tested.

There is also a public health focus on developing novel, effective therapies for delirium and related neurocognitive disorders. One such candidate intervention is caffeine, which optimizes neurocognitive processing (Kuchinke and Lux, 2012; Haller et al., 2013; Chang et al., 2018). In a preliminary clinical trial conducted by our team, surgical participants randomized to intraoperative caffeine demonstrated reduced risk of delirium in the postanesthesia care unit (PACU) concurrent with improved connectivity between frontal and parietal cortices (Vlisides et al., 2021). However, the effects of caffeine on measures of criticality were not tested. Identifying the effects of caffeine on the cortical dynamics underlying delirium could (1) improve neurobiological understanding of cognitive vulnerability and (2) result in a novel, neurobiologically informed intervention for preventing delirium.

The primary objective of this study was to test the relationship between criticality, *via* surrogate electroencephalographic (EEG) measures, and early postoperative delirium. Specifically, this study tested the primary hypothesis that deviations from criticality would be associated with delirium in the PACU. The secondary objective was to determine whether caffeine, administered during surgical closure, would affect measures of criticality in the early postoperative setting given that caffeine has previously been demonstrated to reduce early postoperative delirium (Vlisides et al., 2021).

## Materials and Methods

This was a secondary analysis of a randomized controlled trial that tested the effects of caffeine on pain and neuropsychological recovery after surgery (^1^ NCT03577730, registered 7/5/2018, PI: Vlisides) (Vlisides et al., 2021). The trial was approved by the University of Michigan Medical School Institutional Review Board (Ann Arbor, MI, United States; HUM00135919), and the primary aim of this substudy was to test associations between delirium and surrogate EEG measures of neural criticality. The parent study was a two-arm, parallel trial with a 1:1 allocation ratio (placebo: caffeine). The trial was quadruple-blinded, as patients, clinicians, research team members, and analysts were blinded to the intervention. The original trial protocol, full methodology, adverse events, and protocol deviations are available as previously reported (Vlisides et al., 2021). This manuscript contains a distinct EEG analysis relating to delirium and caffeine administration.

### Patient Population

Adult patients (≥ 18 years of age) presenting for laparoscopic colorectal or gastrointestinal surgery were eligible for the parent trial (Vlisides et al., 2021) and this substudy analysis. Exclusion criteria included emergency surgery, cognitive impairment precluding capacity for informed consent, uncontrolled cardiac arrhythmias, seizure disorders, intolerance or allergy to caffeine, preoperative opioid use, history of diabetes [because dextrose 5% in water (D5W) was the placebo], acute liver failure, pregnancy, breastfeeding, severe visual or auditory impairment (which might hinder cognitive function testing), patients unable to speak English, or enrollment in a conflicting research study.

### Caffeine Intervention and Anesthetic Management

As reported previously (Vlisides et al., 2021), participants were randomized to caffeine citrate (200 mg) or placebo (D5W) *via* blocks of four, stratified by age (≥ 65 years old vs. < 65 years old) and sex. The Michigan Medicine Research Pharmacy prepared intravenous solutions of caffeine citrate (200 mg caffeine equivalent) or D5W per standard pharmacy protocols. The pharmacy was also responsible for the randomization schedule and allocation concealment. On the day of surgery, the study drug was delivered to the main operating room pharmacy, and the drug was then retrieved by a member of the study team. The study drug (40 mL dilution) was then infused over 1 h on a timed infusion pump beginning at surgical closure.

Aside from study drug administration and data collection, no other changes were implemented in the intraoperative or perioperative setting. Anesthetic and surgical management were left at the discretion of the respective care teams, and all standard perioperative procedures were followed per hospital protocols.

### Electroencephalographic Data Acquisition

EEG data were acquired using previously described methods (Vlisides et al., 2021). In brief, data were recorded from a whole-scalp, 16-channel silver/silver chloride electrode system (Cognionics, Inc., San Diego, CA, United States). Measurements were taken for head circumference (nasion to inion) and distance between the preauricular notches was measured for properly aligning the Cz electrode. Data were referenced to the mastoid and sampled at 500 samples/second, and impedances were maintained below 100 kΩ per manufacturer recommendations. Upon completion of the recording session, data were exported to software platforms (as described below) for subsequent analysis.

### Electroencephalographic Data Analysis

Using the MATLAB open source toolbox EEGLAB (Delorme and Makeig, 2004), data were visually inspected for artifact, preprocessed *via* resampling to 250 Hz, and bandpass filtered from 0.5 to 45 Hz. Data were then parsed into 30-s epochs, and all analyses were conducted using a 10-s moving window. Subsequent analyses focused on criticality and neural signal diversity within each of the following frequency bands: delta 0.5 – 4 Hz, theta 4 – 8 Hz, alpha 8 –12 Hz, high alpha 10 – 14 Hz, beta 14 – 25 Hz, and gamma 25 – 45 Hz. EEG measures were analyzed during pre-operative baseline (eyes closed, prior to premedication), intraoperatively, and during PACU recovery (2 min, eyes closed, after PACU admission and initial nurse evaluation).

#### Neural Criticality in the Time and Spatial Domains

Since multichannel EEG data contain spatiotemporal neural information that reflects various brain states, we applied two criticality indicators that emphasize the characteristics of criticality in the time and spatial domains, respectively, as described in the following sections.

#### Criticality in the Time Domain: Autocorrelation of Phase Synchronization

**FIGURE 1:**
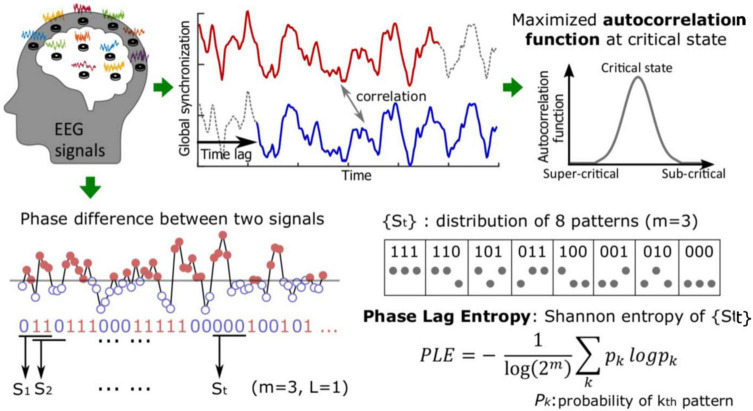
Whole-scalp, 16-channel electroencephalographic (EEG) data were obtained from all participants. Following preprocessing, autocorrelation function and phase lag entropy (PLE) were analyzed from the EEG waveform data. The autocorrelation of global synchronization was calculated with various time lags. For PLE, the phase difference was first calculated and binarized to compute the distribution of the patterns. PLE was then defined with the Shannon entropy of the probability of the patterns (see text for references).

Criticality was assessed using autocorrelation function, a function of time lag referring to the correlation between a time series and its lagged time series, which describes how well the present value of the series relates to past values. This also reflects “temporal memory” of a system, and increasing autocorrelation (i.e., increasing temporal memory) is one of the characteristics of a system that is approaching a critical transition point (Chialvo et al., 2020) (Figure 1). The phenomenon called “critical slowing down” refers to the tendency of a system to take longer to return to equilibrium after a perturbation, which is indicated by an increase in signal variance and autocorrelation (Scheffer et al., 2009). The autocorrelation function of a sequence of order parameters, *r(t)*, with length N, mean μ, and variance *v* is calculated by the following:


$$\mathrm{Autocorrelation}\,\mathrm{Function}\,\left(\mathrm{lag}\right)=\frac{\sum_{t=1}^{N-l\,a\,g}\left(R\,e\,\left[z\,\left(t\right)\right]-\mu \right)\,\left(R\,e\,\left[z\,\left(t+l\,a\,g\right)\right]-\mu \right)}{V},s=1,\ldots ,N/2,$$

where Re[z(t)] is the real part of *z(t)*.


$$z\,\left(t\right)=r\,\left(t\right)\,e^{i\,\Psi \,\left(t\right)}=\frac{1}{N}\,\sum_{j=1}^{N}e^{i\,\theta \,{\left(t\right)}_{j}},$$

where *Ψ(t)* is the average phase at a time *t*. The absolute value *r(t)* = | z(t)| or so-called instantaneous order parameter represents the degree of global phase synchronization at a time *t*. The *r(t)* is equal to 0 when the phases of nodes are uniformly distributed in [0,2π) and 1 when all the nodes have the same phase.

For the EEG analysis, various frequency bands were assessed (0.5 to 45 Hz, with a bin size of 1 Hz) to identify changes in autocorrelation function for global synchronization within each bandwidth. Autocorrelation patterns throughout each frequency band were then presented *via* spectrogram images to illustrate the strength of phase autocorrelation between groups (i.e., delirium vs. no delirium and caffeine vs. no caffeine) and across each frequency band. The primary analysis focused on the alpha band (8 – 12 Hz), given that alpha oscillations are postulated to play a vital role in coordinating spatiotemporally distributed information in the brain during waking consciousness (Linkenkaer-Hansen et al., 2001; Palva et al., 2013; Vlisides et al., 2018).

#### Criticality in the Spatial Domain: Topography of Phase Lag Entropy

Based on empirical and network modeling analysis, functional connectivity appears to resemble structural connectivity at criticality, whereby a distinctive anterior-posterior pattern of partial phase locking (2 – 25 Hz) is observed (Moon et al., 2017; Lee et al., 2019). That is, posterior network nodes with large degree (i.e., more intensive connections) demonstrate large synchronization with connected nodes and, thus, lower phase lag entropy. Conversely, phase lag entropy appears to be higher in frontal regions with peripheral, less strongly connected nodes. This anterior-posterior asymmetry has been observed during baseline waking consciousness and correlates with criticality based on anatomically informed network modeling studies (Lee et al., 2019). This spatial phase lag entropy pattern can thus be used as a practical method to determine distance from criticality during pathologically perturbed states (*via* correlation of topographic phase lag entropy patterns).

Phase lag entropy incorporates the consecutive temporal patterns of the instantaneous phase-lead lag relationship between two signals and quantifies the diversity of phase relationship (Lee et al., 2017) (Figure 1). To calculate phase lag entropy, the phase difference is first binarized; the symbol *s*_*t*_ = 1 if ΔΘ_*t*_ > 0 (the first signal leads the second signal), and *s*_*t*_ = 1 if ΔΘ_*t*_ < 0 (the second signal leads the first signal). The vector *s*_*t*_ is the temporal patterns of phase lead-lag relationship for a given dimension m:


$$S_{t}=\left\{s_{t},s_{t+1},\ldots ,s_{t+{\left(m-1\right)}^{*}\,L}\right\},$$

where m is the length of pattern (e.g., eight patterns with *m* = 3, “000,” “001,” “010,” “100,” “011,” “101,” “110,” and “111”) and the *s*_*t*_ is calculated with time lag *L*.

Phase lag entropy is then calculated by applying the standard Shannon entropy formular to the distribution of the phase difference patterns,


$$P\,h\,a\,s\,e\,l\,a\,g\,e\,n\,t\,r\,o\,p\,y=-\frac{1}{\log\left(2^{m}\right)}\,\sum_{k}p_{k}\,l\,o\,g\,p_{k},$$

where *p*_*k*_ is the probability of *k*th pattern among the possible patterns. *p*_*k*_ is estimated from the fraction of *k*th patterns within all patterns over time. Phase lag entropy is near 0 if a few patterns are dominant, while phase lag entropy ≈ 1 if there is no dominant pattern. In this study, we chose the pattern size of *m* = 3 and *L* = 1 following a similar, previous EEG-based approach (Lee et al., 2019). Phase lag entropy topographs were then constructed by averaging values of from each EEG channel for each group (e.g., placebo, caffeine). Topographic similarity between groups was then compared *via* Pearson correlation between node degrees.

Overall, autocorrelation of phase synchronization and topography of phase lag entropy enable the investigation of surrogate EEG-based measures of criticality based on time and spatial domains, respectively. Results allow for determination of proximity to criticality after pathologic (e.g., delirium) and pharmacologic (e.g., caffeine) perturbations.

### Study Outcomes and Measures

Postanesthesia care unit (PACU) delirium served as the primary outcome for the study. The Confusion Assessment Method (3-min diagnostic version) (Inouye et al., 1990; Marcantonio et al., 2014) was used for assessing delirium. Delirium screens occurred approximately 1 h after PACU arrival by a trained member of the research team. EEG data were collected approximately 20 min after PACU arrival (after anesthetic handoff and PACU nurse assessment). Surrogate EEG measures of neural criticality – alpha autocorrelation function of global synchronization and phase lag entropy topography, as described above – were then evaluated in relation to delirium and caffeine.

### Statistical Analysis

Exploratory data analysis techniques were used to summarize data characteristics, and the Shapiro-Wilk test was used to assess normality of distribution. Levene’s Test for Equality of Variances was also conducted to compare variances between groups given the relatively small – and often unequal – sample sizes. For parametric data, means and standard deviations are presented, and Student’s *t*-test was conducted for comparing means between groups, as Levene’s Test was not significant for any comparisons tested. For non-parametric data, medians and interquartile ranges are presented, and the Mann-Whitney U test was used for group comparisons. P-values less than 0.05 were considered statistically significant. All analyses were conducted using IBM SPSS.

## Results

**FIGURE 2:**
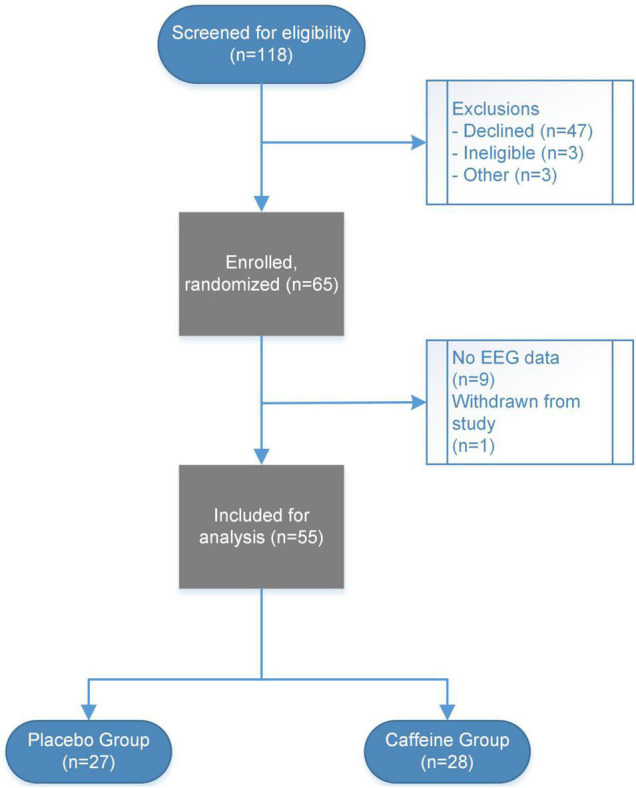
Study flow diagram presented. In total, 118 participants were screened for inclusion, with 65 total participants enrolled and randomized in the parent trial. No EEG data were collected for nine participants, and one participant was withdrawn from the study prior to EEG data collection. EEG and delirium data were thus available for a subset of 55 participants (27 in the placebo group and 28 in the caffeine group).

Study flow is presented in Figure 2. In total, EEG data were analyzed from 55 participants (28 randomized to caffeine and 27 randomized to D5W placebo). Available EEG data were analyzed from 11 delirious participants (four from the caffeine group, seven from the placebo group). For the overall cohort, the mean age was 53 (± 17), 28 (51%) were male sex, median ASA score was 2 (2 – 3), and the majority of participants 49 (89%) presented for laparoscopic colorectal surgery. Complete baseline characteristics are available in Table 1.

**TABLE 1:** Participant characteristics are presented.

	Surgical patients (*n* = 55)
Age, year (IQR)	53 (17)
Male sex, n (%)	28 (51)
Race, n (%)	
White	54 (98)
Asian	1 (2)
Ethnicity, n (%)	
Non-Hispanic	52 (95)
Hispanic	3 (5)
ASA Score, n (IQR)	2 (2 – 3)
Type of surgery, n (%)	
Colorectal	49 (89)
Small intestinal	6 (11)
Comorbidities, n (%)	
Anxiety	6 (8)
Atrial fibrillation	1 (2)
Chronic kidney disease	2 (4)
Chronic obstructive pulmonary disease	1 (2)
Coronary artery disease	1 (2)
Depression	10 (18)
Hypertension	17 (31)
Malignancy	31 (56)
Obstructive sleep apnea	9 (16)
Stroke	1 (2)
Transient ischemic attack	1 (2)

*IQR, interquartile range; ASA, American Society of Anesthesiologists.*

### Delirium, Criticality, and Caffeine

**FIGURE 3:**
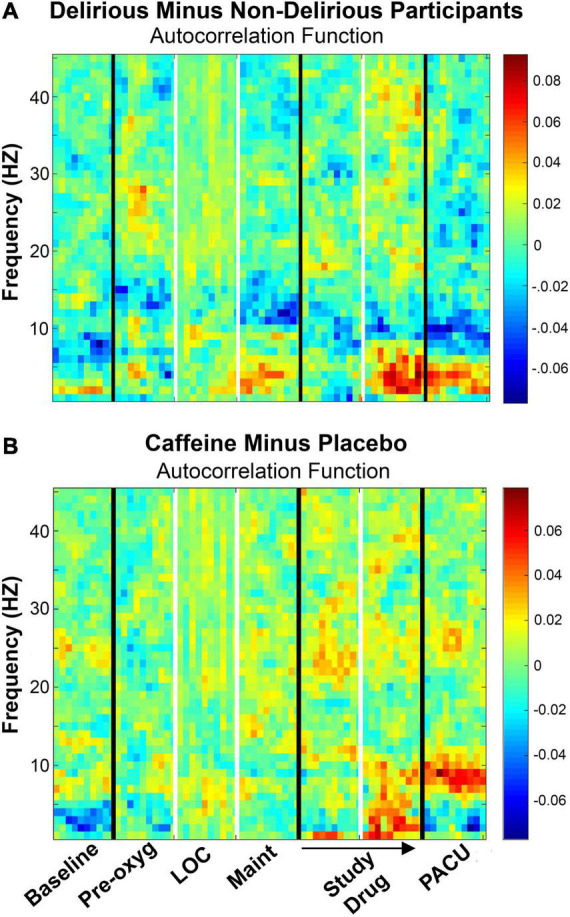
**(A)** Autocorrelation function presented for non-delirious participants (*n* = 42) subtracted from delirious participants (*n* = 11) across study epochs. EEG data were obtained in the PACU after initial admission and nursing evaluation. During PACU evaluation, alpha (8 – 12) and high alpha (10 – 14 Hz) autocorrelation function were significantly reduced in delirious participants, and theta (4 – 8 Hz) autocorrelation function was significantly increased with delirium. **(B)** Autocorrelation function presented for caffeine (*n* = 28) minus dextrose 5% in water placebo (*n* = 27) participants. Participants receiving caffeine demonstrated significantly higher alpha (8 – 12 Hz) autocorrelation function during PACU recovery. Color bars represent median autocorrelation function differences between groups. Pre-oxy, pre-oxygenation; LOC, loss of consciousness; Maint, maintenance anesthesia phase; PACU, postanesthesia care unit; PLE, phase lag entropy.

Autocorrelation spectrograms are presented in Figure 3, which reflect autocorrelation function of global phase synchronization for non-delirious participants subtracted from those experiencing delirium while in the PACU. During PACU recovery, delirious participants demonstrated reduced alpha (8 – 12 Hz) autocorrelation function and increased theta (4 – 8 Hz) autocorrelation function compared to non-delirious participants (Figure 3A). Conversely, those randomized to caffeine demonstrated higher alpha autocorrelation function compared to non-delirious participants (Figure 3B).

In terms of quantitative analysis, PACU alpha autocorrelation function (mean, ± standard deviation) was significantly reduced in delirious participants (0.703 ± 0.034) compared to those without delirium (0.739 ± 0.053) participants (*p* = 0.036). High alpha (10 – 14 Hz) autocorrelation function [median, (interquartile range)] was also significantly lower in delirious participants [0.722 (0.621 – 0.737) vs. 0.750 (0.713 – 0.768); *p* = 0.009] during PACU assessment. Concurrently, PACU theta autocorrelation function was significantly higher in delirious patients (0.756 ± 0.074) compared to patients without delirium vs. (0.710 ± 0.050; *p* = 0.018) (Figure 3A). In terms of caffeine results, alpha autocorrelation function was significantly higher in patients receiving caffeine (0.754 ± 0.045) compared to placebo (0.708 ± 0.046; *p* < 0.001) during PACU recovery (Figure 3B). For the four patients randomized to caffeine who experienced delirium, mean alpha autocorrelation function was low (0.710 ± 0.038) and close to that of the placebo and delirium groups.

### Topography and Phase Signal Diversity

**FIGURE 4:**
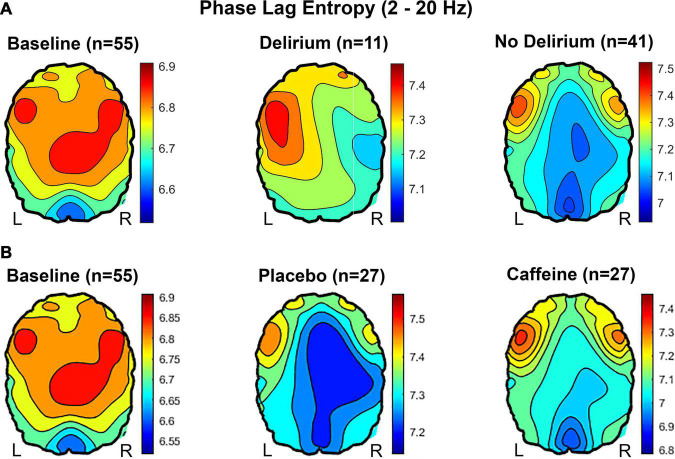
Topography of phase lag entropy (2 – 20 Hz) presented. **(A)** At preoperative baseline, participants demonstrate relatively high frontal and central phase lag entropy compared to posterior regions. This anterior-posterior asymmetry, with relatively high phase lag entropy in anterior regions, appears more preserved in non-delirious participants compared to those with delirium during PACU recovery. **(B)** Relatively low phase lag entropy is present throughout anterior, central, and posterior regions in participants randomized to placebo, whereas relatively high anterior phase lag entropy is present with caffeine during PACU recovery.

Topography of phase lag entropy (2 – 20 Hz) is presented in Figure 4. For the preoperative, baseline state, phase lag entropy was highest in anterior and central regions and relatively low posteriorly (Figures 4A,B, left panels). Postoperatively, phase lag entropy appeared globally higher (see scales, Figure 4), though the anterior-posterior relationship appeared more preserved in non-delirious patients (Figure 4A, right panel), as the node degree correlation to baseline was stronger in non-delirious patients (*r* = 0.837, *p* < 0.001) compared to those with delirium (*r* = 0.553, *p* = 0.05). In terms of caffeine analysis, the anterior-posterior phase lag entropy pattern appeared topographically closer to the baseline state in participants receiving caffeine (Figure 4B), and the correlation to baseline was slightly stronger (*r* = 0.878, *p* < 0.001) compared to those receiving placebo (*r* = 0.718, *p* = 0.006).

**FIGURE 5:**
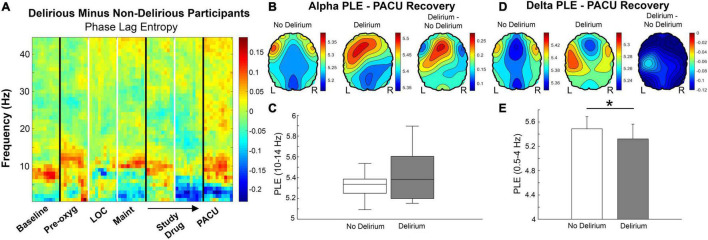
**(A)** Phase lag entropy presented for non-delirious participants (*n* = 42) subtracted from delirious (*n* = 11) participants bandwidths and study epochs. High alpha (10 – 14 Hz) phase lag entropy appears increased during delirium in the PACU based on spectral comparisons (red shading). Conversely, delta (0.5 – 4 Hz) phase lag entropy appears lower with PACU delirium (blue shading). Color bars represent median phase lag entropy differences between groups. **(B)** Alpha phase lag entropy appears increased in anterior regions during delirium. Color bars represent phase lag entropy for the left and middle panels and median difference between groups in the right panel. **(C)** Although high alpha phase lag entropy was higher in patients with delirium, this increase did not reach statistical significance [5.38 (5.20 – 5.60) vs. 5.34 (5.25 – 5.39); *p* = 0.258]. **(D)** Delta phase lag entropy appeared globally reduced during delirium. Color bars represent median phase lag entropy for the left and middle panels and median difference between groups in the right panel. **(E)** Global delta phase lag entropy was significantly reduced from non-delirious to delirious participants (5.49 ± 0.200 vs. 5.32 ± 0.243, respectively; *p* = 0.023). Pre-oxy, pre-oxygenation; LOC, loss of consciousness; Maint, maintenance anesthesia phase; PACU, postanesthesia care unit; PLE, phase lag entropy.

**FIGURE 6:**
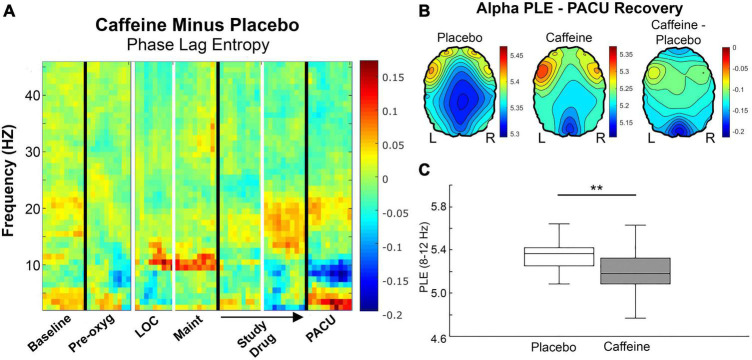
**(A)** Phase lag entropy presented for participants randomized to placebo (*n* = 27) subtracted from those randomized to caffeine (*n* = 28) across bandwidths and epochs. Alpha (8 – 12 Hz) appears reduced with caffeine during postanesthesia care unit recovery (blue shading). Color bars represent median phase lag entropy differences between groups. **(B)** The anterior-posterior alpha phase lag entropy gradient appears diminished in the placebo group compared to caffeine during PACU recovery, and alpha phase lag entropy appears particularly reduced posteriorly in the caffeine group. Color bars represent median phase lag entropy for the left and middle panels and median difference between groups in the right panel. **(C)** Overall, alpha phase lag entropy is relatively lower in the caffeine group [5.18 (5.09 – 5.23)] compared to placebo [5.37 (5.25 – 5.42); *p* = 0.005] during PACU recovery. Pre-oxy, pre-oxygenation; LOC, loss of consciousness; Maint, maintenance anesthesia phase; PACU, postanesthesia care unit; PLE, phase lag entropy.

Phase lag entropy spectrograms were then generated to analyze bandwidth-specific changes with respect to delirium and caffeine. There appeared to be increased alpha phase lag entropy during delirium (Figure 5A). However, the increase during delirium [5.37 (5.19 – 5.65)] was not significant compared to non-delirious participants [5.29 (5.17 – 5.38); *p* = 0.161]. Likewise, there was no significant difference in high alpha phase lag entropy between delirious [5.38 (5.20 – 5.60)] and non-delirious patients [5.34 (5.25 – 5.39); *p* = 0.258; Figure 5C]. Alpha phase lag entropy appeared highest within anterior regions during delirium (Figure 5B). PACU Delta (0.5 – 4 Hz) phase lag entropy also appeared reduced in delirious patients based on spectral analysis (Figure 5A). Mean PACU delta phase lag entropy was 5.49 (± 0.200) in non-delirious patients compared to 5.32 (± 0.243) in delirious patients (*p* = 0.023; Figure 5E). This decreased delta phase lag entropy appeared globally throughout anterior and posterior regions (Figure 5D). Participants randomized to caffeine demonstrated significantly reduced alpha phase lag entropy compared to placebo [5.18 (5.09 – 5.32) vs. 5.37 (5.25 – 5.42); *p* = 0.005, Figures 6A,C]. This reduced alpha appeared localized to the posterior region (Figure 6B, far right panel).

## Discussion

This secondary analysis of a perioperative clinical trial suggests that delirium reflects deviations from neural criticality based on EEG-derived surrogate measures; namely, autocorrelation function of global phase synchronization and topography of phase signal diversity. Additionally, caffeine may shift cortical dynamics toward criticality for supporting cognition, given that caffeine significantly reduced PACU delirium [as reported previously (Vlisides et al., 2021)] concurrent with increased EEG signatures of criticality. As this was a secondary analysis of a clinical trial with a relatively small sample size – and a low-resolution EEG system – the results should be viewed as hypothesis-generating.

It is nonetheless conceivable that delirium reflects disruptions in neural criticality. Inattention is a hallmark feature of delirium, and resting-state criticality, based on neuronal oscillation patterns, correlates with performance on sustained visual attention testing (Irrmischer et al., 2018). Proximity to criticality in the resting state may allow neuronal networks to dynamically shift for meeting sensorimotor system demands (i.e., sustained attention tasks). Deviations from criticality in the resting state could thus predispose to delirium, and other related brain states, by preventing neuronal state transitions and ultimately limiting the available repertoire of brain states available for conscious processing. Indeed, our preliminary data suggest that reductions in neurophysiologic signatures of criticality during eyes-closed, resting state are associated with delirium during PACU recovery. While these are preliminary findings, the results align with human volunteer studies demonstrating an association between critical dynamics, based on neuronal oscillatory patterns in sensorimotor networks, and key cognitive functions (e.g., attention) affected by delirium (Irrmischer et al., 2018).

In this study, a surrogate EEG-based measure of neural criticality – autocorrelation of global alpha rhythm synchronization – was reduced with delirium. Long-range temporal correlation of alpha rhythms (so-called alpha autocorrelation) reflects spatiotemporal neuronal synchronization across different regions of the brain, which may allow for facile coordination of neural processes required for multisensory integration and attendant cognitive demands (Linkenkaer-Hansen et al., 2001; Irrmischer et al., 2018; Vlisides et al., 2018). In fact, resting-state alpha autocorrelation is associated with performance on various executive function tasks (Palva et al., 2013; Irrmischer et al., 2018). Global alpha autocorrelation function in the resting state may thus serve as a candidate biomarker for proximity to neural criticality and, by extension, cognitive capacity. By contrast, theta autocorrelation was significantly increased in patients with delirium. Increasing theta resonance is associated with sleep propensity (Finelli et al., 2000; Meisel et al., 2013), and theta power and alertness have been negatively correlated in human volunteers during sleep deprivation (Meisel et al., 2013). Theta rhythms during delirium may thus represent a proclivity toward slow-wave states that contribute to impaired arousal. Lastly, the magnitude of the differences in autocorrelation function between groups was relatively small (i.e., approximately 0.036 difference in alpha autocorrelation function between delirious and non-delirious participants). This may reflect tight regulation of neural criticality, akin to pH and acid-base regulation, though additional investigation is required to determine the clinical significance of these values.

Topographical analysis of cortical dynamics revealed an asymmetrical anterior-posterior pattern, whereby baseline frontal and central phase lag entropy was relatively higher than posterior regions. This aligns with previous empirical findings from anesthetic-induced altered states of consciousness in humans and results from brain network modeling studies of criticality (Moon et al., 2017; Lee et al., 2019). This combination of high posterior phase synchrony and high anterior diversity of phase relationships is most pronounced at the point of criticality, which may reflect an optimal balance of cortical dynamics for information integration and segregation (Moon et al., 2017; Lee et al., 2019). In this present study, participants demonstrated this anterior-posterior topographical pattern at preoperative baseline, which appeared more preserved in non-delirious participants compared to those experiencing delirium in the PACU (Figure 4). Likewise, participants receiving caffeine also demonstrated increased topographical similarity to baseline compared to those receiving placebo. Bandwidth-specific analyses of phase lag entropy were then conducted, demonstrating a possible trend toward increased alpha phase lag entropy and reduced delta phase lag entropy in delirious patients. These findings stand in direct contrast to the autocorrelation function results, suggesting a shift toward slow-wave synchrony, and, possibly, further from neural criticality.

Caffeine appeared to optimize cortical dynamics concurrent with delirium risk reduction. Patients receiving caffeine demonstrated significantly increased alpha autocorrelation function, which is notable given that alpha autocorrelation function was significantly reduced with delirium. It is noteworthy that, for the four patients receiving caffeine who experienced delirium, alpha autocorrelation was low and close to the mean value of the placebo group. Alpha phase lag entropy was also significantly reduced with caffeine, which may have reflected restoration of temporal correlation of alpha rhythms and, thus, reduced phase signal complexity. The net result of these pharmacodynamic properties of caffeine may be increased proximity to neural criticality and concurrent access to an expanded repertoire of brain states. Indeed, in a related fMRI study of healthy volunteers, caffeine increased resting state brain entropy within key regions that support cognition, such as the lateral prefrontal cortex, default mode network, and visual cortex (Chang et al., 2018). This increased entropy may reflect improved information processing capacity given the expanded spatiotemporal connections observed within these regions. As such, caffeine may serve as a candidate intervention for reducing early postoperative delirium by shifting cortical dynamics toward criticality. However, these findings are preliminary and require additional testing and replication.

There are notable strengths of this study. Multiple strategies (e.g., autocorrelation function, phase lag entropy topography) were used for testing criticality in relation to delirium and caffeine. These criticality measures also provide a rich assessment of spatiotemporal structure of cortical dynamics during surgical recovery. Comparatively, measures of phase-based connectivity [i.e., phase lag index (Stam et al., 2007)] provide a relatively focused, static assessment of phase lead/lag relationships between signals of interest, rather than a comprehensive evaluation of global neuronal oscillatory patterns over a time period of interest. Using multiple, complementary methods also increases convergent validity with respect to criticality analysis. A whole-scalp EEG system was used to capture global cortical dynamics across different regions. Study rigor was also improved by the quadruple-blinded design and independent auditing. In terms of delirium analysis, the Confusion Assessment Method is a widely used, validated assessment for delirium that lends itself to reproducibility.

Conversely, important limitations warrant consideration. This was a substudy analysis of a single-center trial with a limited sample size. The EEG measures described are surrogate measures of criticality and information processing, and a low-resolution system (16-channels) was used for data acquisition and analysis. A high-resolution system will allow for more robust analysis of neural information processing and should be considered for future studies. Participants were not stratified by preoperative cognitive status, and baseline cognitive function could have impacted neurocognitive outcomes. Lastly, the specific Confusion Assessment Method form used [3-min diagnostic version (Marcantonio et al., 2014)], is an abbreviated method that has not yet been formally validated in the early postoperative setting. Neuroimaging (e.g., functional magnetic resonance imaging) can also be considered in the future to address neuroanatomical implications of criticality during delirium and related brain states.

In summary, early postoperative delirium may reflect deviations from neural criticality. Caffeine may restore cortical dynamics closer to criticality concurrent with improved postoperative cognition. A larger-scale study is warranted to further test these relationships.

## Data Availability Statement

The raw data supporting the conclusions of this article will be made available by the authors, without undue reservation.

## Ethics Statement

The studies involving human participants were reviewed and approved by University of Michigan Medical School Institutional Review Board. The patients/participants provided their written informed consent to participate in this study.

## Author Contributions

HK led the data analysis and interpretation, helped draft the initial manuscript, and critically revised the work for intellectually and scientifically important content. AM and JB substantially contributed to study design and data acquisition and helped draft the initial manuscript. UL, GM, and PV contributed to the study conception, design, and interpretation of the data and critically revised the manuscript for intellectual and scientific content. All authors have approved the final manuscript and agreed to be accountable for the content of the work.

## Conflict of Interest

The authors declare that the research was conducted in the absence of any commercial or financial relationships that could be construed as a potential conflict of interest.

## Publisher’s Note

All claims expressed in this article are solely those of the authors and do not necessarily represent those of their affiliated organizations, or those of the publisher, the editors and the reviewers. Any product that may be evaluated in this article, or claim that may be made by its manufacturer, is not guaranteed or endorsed by the publisher.

## Footnotes

1
www.clinicaltrials.gov
